# The relationship between healthy behaviors and health outcomes among older adults in Russia

**DOI:** 10.1186/1471-2458-14-1183

**Published:** 2014-11-19

**Authors:** Anna Selivanova, Jane M Cramm

**Affiliations:** Erasmus School of Economics, Erasmus University Rotterdam, Rotterdam, the Netherlands; Institute of Health Policy and Management, Erasmus University Rotterdam, P.O. Box 1738, 3000, DR Rotterdam, the Netherlands

**Keywords:** Healthy behavior, Self-rated health, Physical activity, Dietary behavior, Alcohol, Smoking, Older population, Russia

## Abstract

**Background:**

Worldwide, populations are aging and the health of elderly individuals is deteriorating. Healthy habits may slow the process of health deterioration, but research investigating relationships between health and various health behaviors is lacking. This study aimed to investigate the relationships between health and health behaviors (alcohol consumption, smoking, dietary behavior, and physical activity) among older men and women in Russia.

**Methods:**

Wave 1 Study on Global AGEing and Adult Health data (2007–2010) collected in the European portion of the Russian Federation, southern federal districts of the European portion of Russia and from the Asian portion of the country were used for this study. Relationships between self-rated health and four risk behavior measures [physical activity, alcohol consumption, smoking, and dietary behavior (fruit and vegetable consumption)] were examined. Analyses controlled for several socioeconomic factors: gender, age, marital status, educational level, area of residence, ethnicity, and employment status. To estimate the effect of healthy behavior on the probability that participants rated their health as very good/good/moderate/bad/very bad, the ordered logit model and average marginal effects were used.

**Results:**

Sufficient physical activity affected self-rated health most significantly in both genders, whereas excessive alcohol consumption had no significant effect. Smoking had explanatory power (being a current smoker decreased the probability of a very good health assessment and increased the probability of a very bad rating compared with being a non-smoker) among men, but not women. Fruit and vegetable consumption had a strong effect on self-rated health among women, but not men.

**Conclusions:**

The results of this study indicate that health behaviors, especially physical activity, are important for the health of Russia’s older population. Smoking behavior had a strong impact on the health of men, whereas fruit and vegetable consumption was a relevant factor for women. Policies promoting smoking reduction and healthy diet should thus target older men and women, respectively.

## Background

Worldwide, aging has become an urgent issue. Researchers predict that the number of people aged ≥65 years in the global population will increase to 1 billion by 2030, which means that 1/8 of the population will be elderly. In this case, the elderly population will outnumber children aged <5 years for the first known time in human history [1].

In Russia, 23.8% of the population was elderly in 2005, and this percentage is expected to increase further [2] *.* By 2030, the proportion of the population aged <60 years is likely to decrease while that of individuals aged ≥60 years is expected to increase. For example, the populations aged 60–64 and 70–74 years are likely to increase by about 500,000 individuals altogether. The population aged >60 years is predicted to increase by more than 850,000 while the overall population in Russia is expected to decrease dramatically by 18 million people [3]. This rapid aging tendency in the Russian population will change family and workforce structures and the pension burden, and increase healthcare demand. This increased demand will, in turn, lead to higher public healthcare expenditures, which may outweigh the need to build additional schools and improve the education system. This situation is highly undesirable, as the lack of an educated younger workforce can have unfavorable economic consequences. To economize on future government healthcare expenditures, investigation of the population’s lifestyle and health habits would be reasonable. Furthermore, as the health of older people is known to deteriorate, efforts must be made to promote active and healthy aging. Some studies have found that transition has a less pronounced impact on health among elderly individuals than in the working-age population. For instance, Leon et al. [4] and Shkolnikov et al. [5] noted that increases and decreases in mortality trends were largest at the ages of 20–45 years, whereas these fluctuations were smallest in older age groups. Some reports have described the healthcare needs of older adults in Russia, but they have not provided a comprehensive, reliable, and clear picture of the health status of this population in different domains [6]. Gurina et al. [6] found that 1.8% (*n* =11) of older adults in their sample had frank malnutrition and 17.3% (*n* =106) were at risk of malnutrition. They also documented a high prevalence of mental problems in elderly Russians. The results emphasize the need for a reallocation of healthcare professionals’ attention and resources away from disease-oriented thinking and toward more functional thinking. Social and environmental factors and economic instability in Russia in the past 20 years may have contributed to the high prevalence of depression in older adults.

A healthy lifestyle can contribute to healthy aging, which in turn decreases healthcare demand. Health deteriorates as people age, increasing disease risk and worsening self-reported health status. Healthy habits can slow this deterioration and may increase the likelihood of positive self-ratings of health in comparison with those expected based on "objective” assessments. Thus, a healthy lifestyle may be a good investment from decision makers’ point of view, as the combined effects of healthy habits may reduce mortality risk [7].

Alcohol and tobacco use are major factors contributing to poor health among men in Russia, whereas obesity and tobacco use are primary factors among men in Western Europe [8]. Alcohol use has been found to contribute more and tobacco use less to poor health among women in the former Soviet Union than among those in Western Europe [8]. Reported alcohol consumption by men and women was strongly associated with mortality from alcohol poisoning; alcohol-associated excesses accounted for 52% of all studied deaths at the ages of 15–54 years and 18% of those at the ages of 55–74 years. Allowance for under-representation of extreme drinkers would further increase the proportions of alcohol-associated deaths [9]. A large gender gap in alcohol consumption exists in Russian society, as this behavior is considered to be masculine [10–13]. Most alcohol-related deaths in Russia occur within the narrow age range of 45–55 years [14]; elderly people may thus be considered to be less prone to alcohol abuse. However, physicians reported growing numbers of alcoholic elderly men after 1994 in response to the social and economic crisis [15]. When examining drinking in the Commonwealth of Independent.

States, Pomerlau et al. [16] found the fewest abstainers in Russia, with this segment of the population increasing with age, usually in association with health deterioration. They reported that Russians traditionally drink spirits, and that the mean drinking frequency tended to decrease with age, reaching the lowest level at the age of ≥65 years [16]. Perlman [17] also found that men aged ≥40 years consumed spirits less frequently than younger men, but that they consumed the most alcohol among age groups when *samogon* (uncertified homemade spirit) consumption was taken into account. Among women, those aged <40 years consumed the most spirits, with an apparent contrast between the majority of women who did not drink or had more Western consumption patterns and a small minority of hazardous drinkers. Younger women drank more than their older counterparts, and female smoking rates also rose most steeply in this age group, suggesting that more young women are adopting risky lifestyles [17]. Gender roles and cultural traditions can determine drinking behavior, whereby women are consistently expected to drink much less than men in terms of preference for strong beverages, drinking frequency, and quantity of alcohol consumed [18].

Smoking is also prevalent in Russia, especially among males [19]. Russia is among the five countries with the highest cigarette consumption per capita. For example, the prevalence of smoking among Russian (56.7%) and Ukrainian (66.8%) men is significantly higher than that among Czech men (38.9%) [20]. Smoking is also prevalent in the elderly male population in Russia. Female smoking is associated with distress and age (elderly women are less likely to smoke than younger females). Roberts et al. [21] reported that the prevalence of smoking in Russia decreased significantly among men, but not women, between 2001 and 2010, although it remained high. The prevalence of smoking among older women was traditionally low, which was not the case for younger women.

Cockerham [22] described dietary behavior as an important unhealthy lifestyle factor in Russia, especially among men. Specifically, fruit and vegetable consumption was insufficient in almost all former Soviet Union countries; the percentage of people consuming fruits daily increased (from 14.9% to 31.2%) and that of people consuming vegetables daily decreased (from 44.6% to 35.5%) during the period 2001–2010, although the quantity of consumption was not examined [23], preventing evaluation using the World Health Organization’s (WHO’s) recommendations. In the late 1990s, poverty was the main reason for insufficient intake of calories, protein, vegetables, and fruits in the elderly population. For example, many pensioners did not receive pensions for extended periods of time during 1996–1999. A report of the Russian Longitudinal Monitoring Service showed that poverty rates doubled and intakes of calories and protein and use of health services and medications declined significantly among affected pensioners. These pensioners were also 5% more likely to die in the 2 years following the crisis [24].

Due to the lack of information and research on physical activity in Russia, the inclusion of this health behavior will shed some light on its effects on self-reported health, which has been neglected to date. Wolinsky and colleagues [25] showed that physical activity and exercise were significantly related to mortality and reduced deterioration in physical function. The benefit of exercise to health outcomes in elderly individuals has been demonstrated across ethnicity, gender, and geographic region. Physical activity and exercise have been shown to reduce the risks of disability and mortality, indicating the need to focus more attention on health promotion programs [26]. In a study comparing self-rated with objectively measured mobility in samples from various countries [27], Russians had the slowest walking speeds and Russian women aged 65–69 years had a high probability (0.68) of reporting difficulty in walking 1 km. Thus, slow walkers had a high probability of reporting mobility difficulty.

Physical activity is closely connected to functional limitation. Among elderly people, functional limitation is the most common factor leading to disability and further dependence on other members of society. Physical function and functional capacity reflect objective health status and are important for well-being and quality of life. Low self-reported physical functioning has been associated with low personal autonomy at the individual level and with a range of health indicators, including mortality [28]. However, Dorynska et al. [28] found that functional limitations were not most common in the sample from Russia, the country with the highest mortality rates.

Cockerham [12, 29] emphasized that health-related habits and social perceptions of health in Russia reflect a communist cultural heritage. Practices embedded in the cultural context of communism include drinking, smoking, poor diet, lack of recreational exercise, and a passive approach to health. A common attitude in Russia is that health depends more on the healthcare system than on individual behavior. This overestimation of the role of the state-owned health care system, which was highly centralized, free, and accessible to all citizens, diminishes the understanding of individual responsibility for personal health and lifestyle.

Most studies of health in Russia have focused on a single health behavior and/or its impact on a particular disease; no study to date has investigated relationships between self-rated health and the four lifestyle factors of alcohol use, smoking, dietary behavior, and physical activity. These factors often coexist and are closely related components of individuals’ lifestyle patterns, which in turn are important predictors of health. The improvement of lifestyle in the aging population by promoting physical activity, healthy diet, and the restriction or cessation of alcohol and tobacco use may alleviate the burdens of healthcare systems by preventing health deterioration in older people*.*

Thus, estimation of the influence of lifestyle on health outcomes, especially in the elderly population, could become a relevant tool for policy makers for the prudent allocation of government healthcare resources. As lifestyle aspects may play major roles in a population’s health, increasing government expenditures on healthy lifestyle promotion may be more efficient. Also, the research depicts which of four risk factors has the most serious effect on self-rated health, which would enable decision makers to determine which aspects of lifestyle need more attention.

### Theoretical background

A large body of evidence has demonstrated that self-reported health assessment has high predictive validity for mortality, physical disability, and chronic disease status. Furthermore, self-assessed health is a stronger predictor of mortality than physician-assessed health [7, 30, 31]. Self-rated health is a valid and extensive measure of health. Obviously, an individual's health status changes over time and a single measure is not capable of reflecting such change. Thus, the importance of self-rated health lies in the stability of its predictive power over time. This feature is also important because a single measure of self-rated health is more easily and less expensively obtained than assessments made by a clinician [7]. In addition, the four health behaviors examined in this study are associated with different disease risks, and disentanglement of these relationships can be difficult. Thus, self-reported health is a good outcome measure that generally depicts how respondents describe their health status.

Grossman’s health production model is the economic framework used in this study [32]. This model describes relationships between health inputs and health outcomes. Individuals are assumed to have a utility function, where utility depends on a stock of health and the consumption of commodities. Unlike medical care, health cannot be purchased directly. It can be produced using time and health-improving efforts (e.g., medical care use). As health behaviors can affect the stock of health, their inputs must be taken into account in the model. Optimality can be reached by making trade-offs between options that increase utility by providing direct satisfaction in the short term and those that increase utility by improving health in the long term. This model can be expressed by the following equations:


where *H* = stock of health, *C* = consumption of non-medical goods and services, *M* = medical care, *L* = lifestyle factors, and *Educ* = level of education.

The influence of health behaviors on health is not immediate. Behaviors such as unhealthy dietary practices can influence current utility, but damage health and reduce health stock in the long term. By contrast, healthy habits such as regular exercise may decrease current utility (e.g., *via* muscle pain) but increase health stock. Furthermore, heterogeneity in health preferences must be taken into account. The investment in health realized by any individual depends on initial endowment and decisions about engaging in healthy behaviors [33].

### Study aims

This study was conducted to investigate relationships between self-rated health and health behaviors (alcohol consumption, smoking, dietary behavior, and physical activity) among older men and women in Russia.

## Methods

### Data source and sample

This study utilized data from the WHO’s Study on Global AGEing and Adult Health (SAGE), which was designed to address the lack of extensive and reliable data on aging and health in low- and middle-income countries, including Russia. A multistage cluster sampling strategy was used in Russia to obtain a nationally representative sample including all seven federal districts. Households were classified into one of two mutually exclusive categories: (1) “50+ households,” from which all persons aged ≥50 years were selected; and (2) “18–49 households,” from each of which one person aged 18–49 years was selected [34, 35]. SAGE Wave 0 samples were randomly revisited in SAGE Wave 1 (2007–2010) in the European portion of the Russian Federation. Wave 1 also included new respondents from southern federal districts of the European portion of Russia and from the Asian portion of the country. Wave 1 data from Russia (3938 respondents aged ≥50 years), collected during face-to-face interviews, were used for the present study. Overall response rates were 79% at the household level and 83% at the individual level [34]; these rates were 80% among those aged 50–59 years and 84% among subjects aged ≥60 years. Ethical approval for the study was obtained from all participants and the SAGE study was approved by the WHO Ethics Review Committee.

### Health behavior measures

This study focused on four behavioral measures of risk: physical activity, alcohol consumption, smoking, and dietary behavior (fruit and vegetable consumption). Physical activity involving sports activities such as jogging, running, swimming, heavy lifting, fitness, gym attendance, and rapid cycling and work activities such as chopping, farm work, and digging with a spade or shovel was classified as vigorous. Activities such as housecleaning, washing clothes by hand, stretching, dancing, gardening, and bicycling at regular pace were classified as moderate. Respondents were asked to report the number of days a week on which they engaged in moderate and vigorous physical activity as part of work, sport, and leisure activities, and the average time spent on these activities per day. The WHO-recommended cutoff point of ≥150 min moderate physical activity, ≥75 min vigorous physical activity, or an equivalent combination per week was considered to constitute sufficient physical activity for elderly people (aged ≥65 years) and other adults (aged 18–64 years) [36]. Asking people about the type, frequency, and duration of physical activity can be a relevant and reliable tool for the evaluation of the level of physical activity in a population. The *physically active* variable was dichotomized as 1 (sufficient; ≥150 min physical activity per week) or 0 (insufficient; <150 min/week) and was derived by calculating the number of minutes of physical activity per day (hours × 60 + minutes) and then multiplying by 7 to obtain a weekly value.

Dietary behavior can also be an important factor in achieving a healthy lifestyle. As we lacked some information about respondents’ dietary behavior, such as calorie/protein intake and types of product most frequently consumed, we used fruit and vegetable consumption as an indicator of healthy eating. WHO guidelines [37] use the threshold value of five servings of fruits and vegetables per day to distinguish healthy from unhealthy diets. The *normal fruit and vegetable consumption* variable was thus dichotomized as 1 (≥5 servings of fruits and vegetables per day) and 0 (fewer than 5 servings/day) and derived by summing the quantities of these foods consumed per day.

Smoking behavior was characterized by asking respondents whether they currently used tobacco products; thus, respondents were classified as current smokers (including daily or less frequent smoking) and non-smokers. The c*urrent smoker* variable was dichotomized as 1 (current smoker) and 0 (non-smoker) accordingly.

In Russia, alcohol consumption may pose the most significant risk among the four factors considered in this study [22, 38]. The most thorough way to assess this behavior is by summing the number of alcoholic drinks respondents report consuming per day during a week. The SAGE data do not specify alcoholic beverage type (e.g., spirits, wine, or beer), but categorize respondents as non-drinkers (0; alcohol consumption on 0 days/week), not heavy drinkers (1; alcohol consumption 1–3 times/week), and heavy drinkers (2; alcohol consumption 4 times/week or more). Maximum alcohol intake for men and women differed among countries, but was most commonly 3 units^a^ per day for men and 2 units per day for women. We used the International Center for Alcohol Policies (ICAP) recommended weekly intakes of 14 units for women and 21 units for men [39], as no clear norm has been established for the Russian Federation. Previous studies, such as those conducted by Cockerham [22, 40], used only the frequency, not the quantity, of alcohol consumption. Nevertheless, it is expected to catch both frequency and quantity, which requires the establishment of daily or weekly cutoff points for alcohol consumption. As cutoff points vary among countries, we used the ICAP recommendations because they are applicable for defining low-risk doses of alcohol for males and females in different countries. Respondents with weekly intakes below the thresholds of 21 units for men and 14 units for women, derived by summing daily intake quantities, were considered to have normal alcohol consumption (coded as 1), and those with intakes above these thresholds were considered to have excessive consumption (coded as 0).

### Outcome measure

Health status was measured *via* self-rated health, which is considered to be a valid and robust measure [7, 30, 31]. Self-rated health was coded as 1 (very good), 2 (good), 3 (moderate), 4 (bad), or 5 (very bad).

### Sociodemographic factors

To investigate the effects of lifestyle factors on self-rated health, several socioeconomic factors (e.g., gender, age, marital status, education, area of residence, ethnicity, and occupation) must be taken into account. Control for gender is important because of differences in dietary behavior (women prefer to eat more fruits, vegetables, and dairy products, whereas men prefer to eat meat and meat products, eggs, and fried food) [41], and smoking and alcohol consumption [38]. The Grossman model also recommends control for age and education level. Health behaviors may also differ according to area of residence (rural *vs*. urban). Religious and cultural preferences can be partially accounted for by including ethnicity in analyses. Marital status and occupation can also affect the willingness and opportunity to lead a healthy lifestyle. For example, people working full time may not have time for exercise and may not be able to eat proper meals in the workplace. Loneliness (being divorced/widowed/single and low social support level) has also been associated with worse health, distressed feelings, and heavier drinking [42]. For these reasons, these sociodemographic factors were included to increase the explained variation and thus the precision of the model:

-  
*Age* – continuous variable (≥50 years)
-  
*Area of residence* – binary variable (1 = urban, 0 = rural)
-  
*Marital status* – transformed variable [1 = never married/separated/widowed (1/4/5), 0 = currently married or cohabiting (2/3)]
-  
*Educational level* – continuous variable indicating full years of completed education
-  
*Ethnicity –* categorical variable (1 = Russian, 2 = Ukrainian/Belorussian, 3 = from nations of Caucasus region (Armenians,Ossetians, Adygeys, Abkhazes,Dagestanis,etc.), 4 = from nations of Volga region (Tatars, Chuvashes, Volga Germans, Maris, etc.), 5 = from nations of Middle Asia (Afghans, Mongols, Kazakhs, Kyrgyzes, Tajikis,etc.), 6 = Altaian, Buryat, or Kalmyk, 7 = other)
-  
*Employment status* – binary variable (1 = currently working, 0 = not working).

### Analysis and model

First, descriptive statistics were calculated to characterize participants’ self-rated health and to determine the number of participants leading healthy lifestyles [non-smokers who ate more than five servings of fruits and vegetables daily, were physically active (average >150 min exercise/week), and had normal alcohol consumption (<2 units/day or 14 units/week for women, <3 units/day or 21 units/week for men)]. An ordered logit model was then used to estimate the effects of healthy behaviors on the probability of having very good/good/moderate/bad/very bad self-rated health. The ordered logit method was chosen because the dependent variable (self-rated health) is ordered and discrete. Probabilities were calculated using the following formula:


where *y* = self-rated health (categorized as *j* =1–5); *x* represents the explanatory variables (age, area of residence, educational level, marital status, ethnicity, employment status, current smoking status, alcohol consumption, physical activity, fruit and vegetable consumption); and τ = thresholds for estimation of latent variable *y*^a^.

To compare magnitudes of the effects of different variables and estimates relative effects must be investigated (i.e. ratio of coefficients), and at the average marginal effects for the variables with a coefficient significantly different from zero. Average marginal effects show how many individuals have a significant marginal effect in the sample given their particular situation and unobserved characteristics [43, 44]. This technique was used to determine which of the four healthy behaviors had the largest and most significant effect.

The following formulas were used to calculate the marginal effects of binary (e.g., sufficient/insufficient physical activity) and continuous (e.g., age) explanatory variables, respectively:


and


Analyses controlled for all sociodemographic factors mentioned above. All analyses were performed using STATA software (version 12 for Windows OS).

## Results

A total of 3938 respondents aged 50–100 (mean, 65) years participated in the survey. The average ages of male and female participants were 63.8 and 65.8 years, respectively, and most participants lived in urban areas. More than 80% of the respondents were of Russian nationality. While more than 50% of females are single only 20% of males are single. The education level was slightly higher among men than women, and more men than women were employed. Table 1 shows descriptive statistics and missing values for all variables. Unadjusted and adjusted (accounting for missing values) percentage rates were calculated.

**Table 1:** **Demographic characteristics of males and females older than 50 in Russia (SAGE Wave 1: 2007–2010)**

Characteristics	Total	Males	Females	χ ^2^	t	p	Missing values	Missing values	Missing values
	n = 3938	n = 1404	n = 2534				Total	Males	Female
Age (years; mean (SD))	65.067(10.18)	63.786(9.83)	65.778(10.31)		84.15	0.001	585	309	276
Urban (yes) (unadjusted)	75.43%(75.43%)	72.20%(72.20%)	77.41%(77.41%)	15.57		≤0.001	0	0	0
Marital status (single) (unadjusted)	43.99%(38.3%)	19.74%(16.18%)	57.43%(51.78%)	520.28		≤0.001	585	309	276
Education (years;mean(SD))	11.054(3.71)	11.319(3.59)	10.905(3.77)		53.19	0.001	724	350	374
Ethnicity (Russian) (unadjusted)	80.17%(65.94%)	75.45%(58.7%)	82.80%(70.35%)	47.61		≤0.001	802(overall ethnicity)	380(overall ethnicity)	422(overall ethnicity)
Ethnicity (Belorussian/Ukranian) (unadjusted)	1.80%(1.48%)	1.73%(1.34%)	1.85%(1.57%)	47.61		≤0.001	-	-	-
Ethnicity (Caucasus) (unadjusted)	12.14%(9.98%)	16.29%(12.68%)	9.82%(8.34%)	47.61		≤0.001	-	-	-
Ethnicity (Volga region) (unadjusted)	3.61%(2.97%)	3.83%(2.98%)	3.48%(2.96%)	47.61		≤0.001	-	-	-
Ethnicity (Middle Asia) (unadjusted)	0.59%(0.49%)	0.60%(0.47%)	0.59%(0.5%)	47.61		≤0.001	-	-	-
Ethnicity (Altaians/Buryats/Kalmyks) (unadjusted)	1.08%(0.89%)	0.90%(0.7%)	1.17%(1%)	47.61		≤0.001	-	-	-
Ethnicity (Others) (unadjusted)	0.62%(0.5%)	1.20%(0.93%)	0.29%(0.25%)	47.61		≤0.001	-	-	-
Working status (Working) (unadjusted)	32.78%(28.22%)	40.17%(32.83%)	28.63%(25.41%)	54.1		≤0.001	629	313	316
Current smoker (yes) (unadjusted)	20.25%(15.38%)	55.12%(33.35%)	5.18%(4.42%)	1100		≤0.001	1086	676	410
Below alcohol norm for males (yes) (unadjusted)	97.40%(78.62%)	97.40%(78.62%)	-	-		-	330	330	-
Below alcohol norm for females (yes) (unadjusted)	99.60%(88.74%)	-	99.60%(88.74%)	-		-	306	-	306
Physically active (yes) (unadjusted)	65.27%(64.79%)	61.14%(60.69%)	67.79%(67.28%)	20.61		≤0.001	29	11	18
Sufficient fruits and vegetables consumption (yes) (unadjusted)	22.74%(15.85%)	21.79%(13.96%)	23.25%(17%)	0.8661		0.352	1369	615	754
Self-reported health (mean; (SD))	3.173(0.67)	3.057(0.69)	3.237(0.66)		73.45	≤0.001	599	315	284

The survey revealed the following health behavior characteristics of the elderly Russian population (Table 1). More men (55.12%) than women (5.18%) were current smokers. The majority of the sample had prudent alcohol intake, with only 2.60% and 0.4% of men and women, respectively, reporting excessive alcohol consumption. Fruit and vegetable consumption was insufficient for both genders; only 21.79% of men and 23.25% of women consumed more than five servings of fruits and vegetables daily. More than 65% of the population (61.14% of men, 67.79% of women) was sufficiently physically active.

The results of ordered logit models are presented in Tables 2 and 3. The magnitudes of the result coefficients were obtained by using average unweighted marginal effects, as the ordered models provided information only about the sign and significance of the variables. For both genders, older age decreased the probability of very good and increased the probability of very bad self-rated health while current employment and sufficient physical activity positively affected self-rated health. On average, the probability of good self-rated health was 13.6 and 2.8 percentage points higher among male and female participants, respectively, who engaged in ≥30 min physical activity per day than among those reporting no physical activity, ceteris paribus at the 5% confidence level. Excessive alcohol consumption had no significant effect on self-rated health among men or women in univariate or ordered logit analysis.

**Table 2:** **Relationships between sociodemographic factors, health behaviors and health outcomes among older males in Russia (SAGE Wave 1: 2007–2010)**

	Very good health	Good health	Moderate health	Bad health	Very bad health
	Coefficient (95% confidence interval)	Coefficient (95% confidence interval)	Coefficient (95% confidence interval)	Coefficient (95% confidence interval)	Coefficient (95% confidence interval)
Age	-0.0002(-0.0005; 0.00003)	-0.0029*(-0.005;-0.0003)	-0.00002(-0.0005; 0.0004)	0.003*(0.0004; 0.006)	0.0002(-0.00003; 0.0003)
Urban	-0.000039(-0.003; 0.003)	-0.0005(-0.044; 0.043)	-4.01e-06(-0.0003; 0.0003)	0.0005(-0.045; 0.046)	0.00003(-0.002; 0.002)
Marital status (single)	0.001(-0.003; 0.005)	0.0143(-0.036; 0.065)	-0.0004(-0.004; 0.003)	-0.014(-0.063; 0.035)	-0.0007(-0.003; 0.002)
Education	0.00016(-0.0003; 0.0006)	0.002(-0.004; 0.008)	.0000185(-0.0003; 0.00036)	-0.002(-0.008;0.004)	-0.0001(-0.0004; 0.0002)
Ethnicity (Belorussian/Ukranian)	0.0008(-0.01; 0.012)	0.0116(-0.141; 0.165)	-0.00006(-0.008; 0.0075)	-0.012(-0.16; 0.138)	-0.0005(-0.007; 0.006)
Ethnicity (Caucasus)	0.002(-0.003; 0.007)	0.031(-0.029; 0.092)	-0.0024(-0.013; 0.008)	-0.03(-0.08;0.024)	-0.001(-0.004;0.001)
Ethnicity (Volga region)	-0.001(-0.006; 0.004)	-0.0175(-0.096;0.061)	-0.002(-0.018; 0.0136)	0.0199(-0.07; 0.11)	0.001(-0.004;0.006)
Ethnicity (Middle Asia)	0.036(-0.06; 0.13)	.264(-0.111;0.64)	-0.148(-0.5;0.2)	-0.147*(-0.26;-0.034)	-0.005(-0.01; 0.0002)
Ethnicity (Altaians/Buryats/Kalmyks)	0.01(-0.016; 0.038)	0.120(-0.108; 0.348)	-0.038(-0.167; 0.091)	-0.09(-0.21;0.03)	-0.004(-0.009; 0.001)
Ethnicity (Others)	-0.0076*(-0.014; -0.0017)	-0.1409***(-0.189; -0.093)	-0.22(-0.461; 0.022)	0.033**(0.097; 0.57)	0.037(-0.02;0.096)
Working status (Working)	0.008*(0.0015; 0.0146)	0.125***(0.075; 0.175)	-0.0034(-0.025; 0.018)	-0.12***(-0.17; -0.08)	-0.005*(-0.009; -0.0004)
Current smoker (yes)	-0.0045*(-0.009; -0.00009)	-0.061** (-0.103; -0.02)	0.0009(-0.009; 0.01)	0.062**(0.02;0.102)	0.003(-0.0002; 0.006)
Below alcohol norm for males (yes)	0.0017(-0.005; 0.008)	0.023(-0.072; 0.118)	0.002(-.017; 0.022)	-0.026(-0.14; 0.089)	-0.001(-0.008; 0.005)
Physically active (yes)	0.008**(0.002; 0.014)	0.136***(0.104;0.169)	0.0866***(0.036;0.137)	-0.22***(-0.29;-0.15)	-0.01*(-0.02;-0.0009)
Sufficient fruits and vegetables consumption (yes)	0.0027(-0.0015; 0.007)	0.036(-0.013;0.084)	-0.002(-0.01; 0.006)	-0.034(-0.078; 0.009)	-0.0016 (-0.004; 0.0008)
Overall model evaluation Wald test					
Chi(2)	169.93				
DF	15				
P-value	p≤0.001				
R²	0.1132				

*p ≤ 0.05, **p ≤ 0.01, ***p ≤ 0.001.

**Table 3:** **Relationships between sociodemographic factors, health behaviors and health outcomes among older females in Russia (SAGE Wave 1: 2007–2010)**

	Very good health	Good health	Moderate health	Bad health	Very bad health
	Average marginal effect (95% confidence interval)	Average marginal effect (95% confidence interval)	Average marginal effect (95% confidence interval)	Average marginal effect (95% confidence interval)	Average marginal effect (95% confidence interval)
Age	-0.00007(-0.0001; 0.00001)	-0.003***(-0.004; -0.002)	-0.004***(-0.005; -0.0027)	0.006***(0.004; 0.008)	0.001***(0.0006; 0.0014)
Urban	0.00008(-0.0003; 0.0005)	0.0037(-0.015;0.02)	0.005(-0.022; 0.032)	-0.008(-0.047; 0.032)	-0.0013(-0.008; 0.005)
Marital status (single)	-0.00012(-0.0005; 0.00025)	-0.006(-0.02;0.01)	-0.008(-0.03;0.014)	0.011(-0.02; 0.044)	0.002(-0.003; 0.007)
Education	0.0001(-0.00002; 0.0002)	0.005***(0.0026; 0.007)	0.007***(0.004; 0.01)	-0.01***(-0.015; 0.0056)	-0.002***(-0.003; -0.0008)
Ethnicity (Belorussian/Ukranian)	-0.0007(-0.0018; 0.0003)	-0.034(-0.069; 0.0009)	-0.068(-0.166;0.03)	0.086(-0.02; 0.19)	0.018(-0.01;0.046)
Ethnicity (Caucasus)	0.00006(-0.0006; 0.0007)	0.0025(-0.026; 0.03)	0.003(-0.03;0.04)	-0.005(-0.06;0.05)	-0.0008(-0.01; 0.008)
Ethnicity (Volga region)	-0.0002(-0.001; 0.0006)	-0.009(-0.045; 0.026)	-0.014(-0.07;0.044)	0.02(-0.06;0.1)	0.003(-0.011; 0.018)
Ethnicity (Middle Asia)	-0.0006(-0.002; 0.001)	-0.03(-0.1;0.04)	-0.059(-0.247;0.13)	0.075(-0.136;0.29)	0.015(-0.037; 0.067)
Ethnicity (Altaians/Buryats/Kalmyks)	0.0006(-0.0015; 0.0026)	0.024(-0.05;0.1)	0.025(-0.038;0.09)	-0.043(-0.169;0.08)	-0.006(-0.02;0.01)
Ethnicity (Others)	0.0009(-0.0046; 0.0064)	0.038(-0.16;0.24)	0.035(-0.08;0.15)	-0.06(-0.35;0.22)	-0.009(-0.04;0.026)
Working status (Working)	0.002(-0.0003; 0.004)	0.098***(0.069;0.12)	0.1***(0.076;0.124)	-.0177***(-0.2; -0.14)	-0.02***(-0.03;-0.014)
Current smoker (yes)	0.0004(-0.0006; 0.0013)	0.015(-0.02;0.053)	0.018(-0.02; 0.057)	-0.03(-0.097; 0.038)	-0.005(-0.015; 0.005)
Below alcohol norm for females (yes)	0.001(-0.0003; 0.003)	0.06**(0.02;0.1)	0.19(-0.06;0.448)	-0.19*(-0.38; -0.014)	-0.063(-0.18; 0.0546)
Physically active (yes)	0.0006(-0.0001; 0.001)	0.028***(0.013;0.044)	0.05**(0.017;0.082)	-0.067***(-0.1;-0.026)	-0.01**(-0.02;0.004)
Sufficient fruits and vegetables consumption (yes)	0.0007(-.0002; 0.0016)	0.03**(0.01;0.049)	0.035***(0.015;0.055)	-0.057***(-0.09; -0.02)	-0.009***(-0.014; 0.003)
Overall model evaluation Wald test					
Chi(2)	400.91				
DF	15				
P-value	p≤0.001				
R²	0.1137				

*p ≤ 0.05, **p ≤ 0.01, ***p ≤ 0.001.

The effects of some healthy behaviors differed between men and women. Smoking had explanatory power (being a current smoker decreased the probability of very good health and increased the probability of very bad health compared with being a non-smoker) for men, but not for women. On average, smoking was associated with a 6.2–percentage point increase in the probability of bad health assessment and a 6.1–percentage point reduction in the probability of good health assessment among men, ceteris paribus at a 5% significance level. Also, men whose ethnicity was categorized as 'other' were less likely to assess their health as good or very good and more likely to assess it as bad when compared with ethnic Russians. In contrast, ethnicity was insignificant for females in this sample.

Sufficient fruit and vegetable consumption and higher educational level increased the probability of very good self-rated health and decreased that of very bad health among women, but not men. On average, sufficient fruit and vegetable consumption increased the probability of good health by 3.0 percentage points and decreased that of bad health by 5.7 percentage points among women, ceteris paribus at a 5% significance level.

No strong correlation among health-related variables was observed, with the strongest correlation noted between weekly alcohol consumption and smoking behavior (0.2627). Multicollinearity was assessed using a correlation matrix. Sociodemographic factors showed stronger correlation, primarily associated with age. The strongest correlation was observed between employment status and age (-0.55), indicating the absence of multicollinearity (Table 4). Secondly, the *F*-test for joint significance produced very high *F* values indicating joint significance (and thus no multicollinearity) of all included variables, specifically age and employment status*.*

**Table 4:** **Correlations between variables (SAGE Wave 1: 2007–2010)**

	Age	Urban	Marital (single)	Education	Ethnicity	Working	Current smoker	Alcohol per week	Physical activity	Fruits and vegetables
Age	1									
Urban	0.1013	1								
Marital (single)	0.3149	0.1203	1							
Education	-0.4299	0.1047	-0.1666	1						
Ethnicity	-0.1187	-0.2683	-0.0755	-0.0131	1					
Working	-0.5525	-0.0259	-0.1882	0.3663	0.0910	1				
Current smoker	-0.2025	-0.0368	-0.1807	0.0294	0.0013	0.1176	1			
Alcohol per week	-0.1052	-0.0395	-0.0545	0.0219	-0.0052	0.0420	0.2627	1		
Physical activity	-0.2416	0.0239	-0.0866	0.1664	-0.0555	0.1747	0.0359	0.0297	1	
Fruits and vegetables	-0.0778	-0.0091	-0.0289	0.0690	0.0433	0.0813	-0.0706	-0.0235	-0.035	1

## Discussion

This study aimed to investigate relationships between self-reported health and health behaviors (alcohol consumption, smoking, dietary behavior, and physical activity) among older men and women in Russia. The results show significant differences in health-related behaviors between men and women in this sample: women smoked less, consumed less alcohol, were more physically active, and consumed more fruits and vegetables than did men. In addition, women with such healthy lifestyles reported better health than men on average, although mean self-rated health was poorer among women than among men, in agreement with the findings of many previous studies [45–48].

Physical activity was found to strongly influence self-reported health among elderly men and women in Russia. Few studies have investigated the effects of physical activity in Russian adults. More than 65% of participants in this sample met the WHO recommendation of ≥30 min physical activity per day, five times per week, for elderly individuals [36]. Women were more physically active than men, which may be explained by cultural norms and traditions leading women to engage more frequently than men in moderate activities such as housekeeping, shopping, and gardening [49–51]. Sufficient physical activity was associated with higher probabilities of good or moderate self-rated health among women and moderate to very good health among men, and ≥30 min activity/day increased the probability of good health by 13.6 percentage points for men and 2.8 percentage points for women. Thus, policy makers should implement measures promoting physical activity among adults of both genders, such as the development of more parks, special sports grounds and centers, and special sports competitions or events for adults [52–54]. Further investigation of the effects of physical activity on health among adults in Russia should use a wider range of physical activity measures and dimensions to more thoroughly characterize activity duration, frequency, type, and intensity.

The results of this study indicated that alcohol consumption had no significant effect on self-reported health among men or women. The majority of participants were considered to have normal alcohol intake according to gender-specific norms; only 2.6% of men and 0.4% of women reported excessive alcohol consumption, indicating that heavy drinking is not a common problem in the older population in Russia. Cockerham found that alcohol addiction was a major problem among Russians in the 1990s, but he examined the entire adult population, including younger people. Moreover, Cockerham et al. [55] and Abbott et al. [56] conducted studies in the late 1990s and early 2000s, when the political and economic situation was unstable because of the economic crisis and default of 1998. The present, more economically stable situation is likely to be associated with the reduced frequency and severity of alcoholism, as indicated by the results of the present study. In addition, Neufeld and Rehm [57] found that changes in the Russian alcohol policy (including the regulation of alcohol production and sales) initiated in 2006 as part of the long-term strategy to reduce alcohol-related harm reduced total consumption and mortality. These policy changes were in effect during the period of SAGE Wave 1 data collection, and likely contribute to explaining the low percentage of heavy drinking reflected in these data.

The effects of smoking and fruit and vegetable consumption on self-rated health also differed between men and women in this sample; smoking had a significant negative effect among men, but not women, and fruit and vegetable consumption had a significant positive effect among women, but not men. The smoking results could be explained by the low percentage of female relative to male smokers in the sample; only 5% of women were current smokers, which could have limited the ability to obtain significant results. Among men, smoking was associated with a 6.2–percentage point increase in the probability of poor self-rated health and a 6.1–percentage point decrease in the probability of good health. In determining target audiences, policy makers should thus be aware that anti-smoking policies can have strong impacts on elderly men, but may have little effect on elderly women.

Although fruit and vegetable consumption had a significant effect on the self-rated health of elderly women, only 22.74% of the total population consumed sufficient amounts of these foods, in agreement with most previous findings [23, 24]. Among women, sufficient consumption was associated with a 3–percentage point increase in the probability of good health and a 5.7–percentage point decrease in the probability of poor health. Hinote and colleagues [58] reported a similar positive effect, finding that higher reported levels of distress negatively affecting self-rated health were associated with less frequent consumption of foods such as vegetables and fruit. Older respondents and those who are unmarried, divorced, or widowed tend to report significantly higher levels of distress and less consumption of fruits and vegetables, so the relationships between the level of distress and the consumption of fruit and vegetables remain strong and statistically significant. The insignificance of this variable among men can be explained by gender differences in dietary preferences; women tend to consume more fruits and vegetables, cereals, and dairy products, whereas men are more likely to eat meat, eggs, beer, and fast food [41, 59].

Sociodemographic characteristics also affected the self-rated health of both genders in this study. Age was associated with worse health, in agreement with most previous findings, including those of WHO and SAGE reports. In this sample, single status had no significant effect compared with married/cohabiting status among men or women. Previous studies have suggested that married or cohabiting people are more likely to have better health and use less formal and informal care than are people who live alone [60–62]. This difference might be explained by the greater importance of children’s and grandchildren’s support and care to maintain older parents’/grandparents’ health relative to being married or cohabiting [63, 64]. The results of this study with respect to ethnicity largely confirm those of Karlsen and Nazroo [65], who found that ethnic identity was not related to health. However, we found that men who selected the “other” category of ethnicity had significantly worse health than did Russians; this ethnic categorization was associated with a reduction of more than 14 percentage points in the probability of good self-reported health and 3.3 percentage points increase in that of poor health. This negative assessment could be explained by ethnic minorities’ feelings of loneliness or not belonging in society [65–67]. Contrary to our expectation, education level had no significant effect on self-rated health among men and a significant but small effect among women; 10 additional years of education increased the probability good self-rated health by only 5 percentage points. Current employment was associated with a higher probability of good health assessment among men and women, reflecting the ability of only people in good health to continue working after the age of 50 years. Employment also provides income, allowing individuals to purchase high-quality products (including medications) and services, and thereby maintain better health.

Several limitations of this study should be recognized. The most important limitation is the cross-sectional nature of the study. Longitudinal data would allow causal inference; the data used in the present study allowed only the identification of associations, which is an important step, especially because the relationships of four (rather than one or two) health behaviors to health among elderly individuals were analyzed together. The availability of new waves of SAGE data will enable the investigation of changes in health behavior over time and their effects on the health of Russia’s elderly population. The analysis showed that physical activity was the strongest predictor of adults’ health, which reduced the explanatory power of the other independent variables. Nevertheless, the insignificance of alcohol intake may be due to the spreading or joint effects of other variables, as all four health behaviors were analyzed together. If the study were focused on smoking or alcohol consumption alone, these variables would have stronger predictive power. Moreover, the bias described by Zaridze et al. [68], who found that Russian adults, particularly men, are at high risk of premature death and that alcohol (particularly vodka) was a major determinant of mortality, may have influenced the effect of alcohol consumption in the present study. In addition, self-reported alcohol consumption can vary from year to year; for example, formerly heavy drinkers may report current low consumption due to deteriorating health. Heavy drinking before the age of 50 years could have influenced current health among study participants reporting current low consumption. Such fluctuations in alcohol consumption over time may have biased the results, which we could not control due to the lack of panel data. Another potential bias is under-reporting of drinking among men and women. Zaridze et al. [68] and Bobrova et al. [18] found that the quantity of alcohol consumption reported by an individual did not always coincide with that reported by family members. Women tend to under-report alcohol intake to a larger extent than men, which may lead to overestimation of the gender gap in drinking [18]. Another limitation closely related to the gender gap is the potential lack of clinical relevance of gender differences due to CI96% overlap, which indicates that there is no statistically significant differences between genders for some variables. In addition, this study may have been affected by endogeneity and reverse causality. Endogeneity is a potential issue, as not only can physical activity influence self-rated health, but health can also influence the ability to be physically active. Relationships between health and health behaviors are expected to be dynamic, and longitudinal data will allow examination of these relationships over time. In addition, all variables used in this study were self-reported. To address this limitation, future studies should seek to identify suitable and convincing instrumental variables and to investigate objective as well as subjective health behaviors and outcomes (e.g., using walking, lung function, grip strength tests; biomarker data). Another limitation was the lack of rural residents in the study sample, which compromised our ability to precisely estimate the effect of area of residence. Furthermore, dietary behavior and diet cannot be fully represented by fruit and vegetable consumption; additional factors, such as calorie, protein, and fat intake; types of product consumed; and frequency and size of meals should be accounted for. Consideration of all aspects of eating preferences would provide more opportunities to draw conclusions about the effects of healthy eating on health in both genders, and the usefulness of this factor for policy making.

## Conclusions

The results of this study indicate that health behaviors, especially physical activity, have important effects on the health of Russia's older population. Smoking behavior has a large impact on the health of older men, whereas fruit and vegetable consumption is a relevant factor among older women. To make the best use of available financial resources and improve the efficiency of program implementation, policies promoting smoking reduction and healthy diet should thus target older men and women, respectively. Gender differences in dietary behavior should be explored in greater detail to guide policy making and identify appropriate target audiences to improve health in older men and women.

## Endnote

^a^A unit of alcohol is a measure of the volume of pure alcohol in an alcoholic beverage (10 ml or 8 g ethanol). For example, 175 ml 12% alcohol by volume (ABV) wine contains 2 units of alcohol, half a pint (284 ml) standard 3.5%–5.5% ABV beer contains 1.5 units, and 25 ml 40% ABV spirits contain 1 unit of alcohol.

## References

[1] National Institute on Aging. Growing Older in America (2007). The Health and Retirement Study.

[2] Gavrilova NS, Gavrilov LA, Uhlenberg P (2009). Rapidly aging populations: Russia/Eastern Europe. International Handbook of Population Aging.

[3] U.S. Census Bureau (2007). International Programs.

[4] Leon DA, Chenet L, Shkolnikov VM, Zakharov S, Shapiro J, Rakhmanova G, Vassin S, McKee M (1997). Huge variation in Russian mortality rates 1984–94: artefact, alcohol, or what?. Lancet.

[5] Shkolnikov V, McKee M, Leon DA (2001). Changes in life expectancy in Russia in the mid-1990s. Lancet.

[6] Gurina NA, Frolova EV, Degryse JM (2011). A roadmap of aging in Russia: the prevalence of frailty in community-dwelling older adults in the St. Petersburg district–the “Crystal” study. J Am Geriatr Soc.

[7] Mossey JM, Shapiro E (1982). Self-rated health: a predictor of mortality among the elderly. Am J Public Health.

[8] World Health Organization (2002). World Health Report.Reducing risks, promoting healthy life.

[9] Zaridze D, Brennan P, Boreham J, Boroda A, Karpov R, Lazarev A, Konobeevskaya I, Igitov V, Terechova T, Boffetta P, Petoc R (2009). Alcohol and cause-specific mortality in Russia: a retrospective case–control study of 48,557 adult deaths. Lancet.

[10] Segal BM (1990). The Drunken Society: Alcohol Abuse and Alcoholism in the Soviet Union: A Comparative Study.

[11] Cockerham WC (1997). The social determinants of the decline of life expectancy in Russia and Eastern Europe: a lifestyle explanation. J Health Soc Behav.

[12] Cockerham WC (1999). Health and Social Change in Russia and Eastern Europe.

[13] Van Gundy K, Schieman S, Kelley M, Rebellon C (2005). Gender orientations and alcohol use among Moscow and Toronto adults. Soc Sci Med.

[14] Gavrilova NS, Gavrilov LA, Semyonova VG, Evdokushkina GN, Ivanova AE, Pridemore WA (2005). Patterns of Violent Crime in Russia. Ruling Russia: Law, Crime, and Justice in a Changing Society.

[15] Gafarov VV, Pak VA, Gagulin IV, Babina TD (2003). Ten year dynamics of attitude to health problems in male population of Novosibirsk. Ter Arkhiv.

[16] Pomerleau J, McKee M, Rose R, Haerpfer CW, Rotman D, Tumanov S (2005). Drinking in the commonwealth of independent states–evidence from eight countries. Addiction.

[17] Perlman FJA (2010). Drinking in transition: trends in alcohol consumption in Russia 1994–2004. BMC Public Health.

[18] Bobrova N, West R, Malyutina D, Malyutina S, Bobak M (2010). Gender differences in drinking practices in middle aged and older Russians. Alcohol Alcohol.

[19] Kislitsyna O, Stickley A, Gilmore A, McKee M (2010). The social determinants of adolescent smoking in Russia in 2004. Int J Public Health.

[20] World Health Organization Regional Office for Europe (2007). The European Tobacco Control Report.

[21] Roberts B, Gilmore A, Stickley A, Rotman D, Prohoda V, Haerpfer C, McKee M (2012). Changes in smoking prevalence in 8 countries of the former Soviet Union between 2001 and 2010. Am J Public Health.

[22] Cockerham WC (2000). Health lifestyles in Russia. Soc Sci Med.

[23] Abe SK, Stickley A, Roberts B, Richardson E, Abbott P, Rotman D, McKee M (2013). Changing patterns of fruit and vegetable intake in countries of the former Soviet Union. Public Health Nutr.

[24] Jensen RT, Richter K (2004). The health implications of social security failure: evidence from the Russian pension crisis. J Public Econ.

[25] Wolinsky FD, Stump TE, Clark DO (1995). Antecedents and consequences of physical activity and exercise among older adults. Gerontologist.

[26] Lee Y, Lee KJ, Han GS, Yoon SJ, Lee YK, Kim CH, Kim JL (2002). The development of a physical functioning scale for community-dwelling older persons. J Prev Med Public Health.

[27] Capistrant BD, Glymour MM, Berkman LF (2014). Assessing mobility difficulties for cross-national comparisons: results from the World Health Organization Study on Global AGEing and Adult Health. J Am Geriatr Soc.

[28] Dorynska A, Paja A, Kubinova R, Malyutina S, Tamosiunas A, Pikhart H, Peasey A, Nikitin Y, Marmot M, Bobak M (2012). Socioeconomic circumstances, health behaviours and functional limitations in older persons in four Central and Eastern European populations. Age Ageing.

[29] Cockerham WC, Hinote BP, Cockerham GB, Abbott P (2006). Health lifestyles and political ideology in Belarus, Russia, and Ukraine. Soc Sci Med.

[30] Idler EL, Benyamanini Y (1997). Self-rated health and mortality: a review of twenty-seven community studies. J Health Soc Behav.

[31] Idler EL, Kasl SV (1995). Self-ratings of health: do they predict change in functional ability?. J Gerontol B Psychol Sci Soc Sci.

[32] Grossman M (1972). On the concept of health capital and the demand for health. J Polit Econ.

[33] Humphreys BR, McLeod L, Ruseski JE (2014). Physical activity and health outcomes: evidence from Canada. Health Econ.

[34] Maximova T (2013). Russian Federation - Study on Global Ageing and Adult Health-2007/10, Wave 1.

[35] Kowal P, Chatterji S, Naidoo N, Biritwum R, Fan W, Lopez Ridaura R, Maximova T, Arokiasamy P, Phaswana-Mafuya N, Williams S, Snodgrass JJ, Minicuci N, D’Este C, Peltzer K, Boerma JT, SAGE Collaborators (2012). Data resource profile: the World Health Organization Study on global AGEing and adult health (SAGE). Int J Epidemiol.

[36] World Health Organization (2010). Global Recommendations on Physical Activity for Health.

[37] World Health Organization (2004). Global Strategy on Diet, Physical Activity and Health.

[38] Cockerham WC, Hinote BP, Abbott P (2006). Psychological distress, gender, and health lifestyles in Belarus, Kazakhstan, Russia, and Ukraine. Soc Sci Med.

[39] International Center for Alcohol Policies (ICAP) (2003). International Drinking Guidelines for Alcohol.

[40] Cockerham WC (2007). Health lifestyles and the absence of the Russian middle class. Sociol Health Illn.

[41] Turrell G (1997). Determinants of gender differences in dietary behavior. Nutr Res.

[42] Stickley A, Koyanagi A, Roberts B, Richardson E, Abbott P, Tumanov S, McKee M (2013). Loneliness: its correlates and association with health behaviours and outcomes in nine countries of the former Soviet Union. PLoS One.

[43] Carro JM, Traferri A (2009). Correcting the Bias in the Estimation of a Dynamic Ordered Probit With Fixed Effects of Self-Assessed Health Status. Economics Working Papers we09402.

[44] Fernández-Val I, Savchenko Y, Vella F (2013). Evaluating the role of individual specific heterogeneity in the relationship between subjective health assessments and income. IZA DP.

[45] Lucky S, Arokiasamy P, Singh PK, Rai RK (2013). Determinants of gender differences in self-rated health among older population. Evidence from India. SAGE Open.

[46] Demirchyan A, Petrosyan V, Thompson ME (2012). Gender differences in predictors of self-rated health in Armenia: a population-based study of an economy in transition. Int J Equity Health.

[47] Voormann R, Helemae J (2013). A comparative analysis of gender differences in self-rated health: is the Baltic Sea frontier of the East–west health divide in Europe?. Filisofija Sociologija.

[48] Carlson P (2001). Risk behaviors and self rated health in Russia 1998. J Epidemiol Community Health.

[49] Bird CE (1999). Gender, household labor, and psychological distress: the impact of the amount and division of housework. J Health Soc Behav.

[50] Bianchi SM, Milkie MA, Sayer LC, Robinson JP (2000). Is anyone doing the housework? Trends in the gender division of household labor. Soc Forces.

[51] Cubbins LA, Szaflarski M (2001). Family effects on self-reported health among Russian wives and husbands. Soc Sci Med.

[52] Green M (2007). Governing under advanced liberalism: sport policy and the social investment state. Policy Sci.

[53] Bedimo-Rung AL, Mowen AJ, Cohen DA (2005). The significance of parks to physical activity and public health. A conceptual model. Am J Prev Med.

[54] Mytton OT, Townsend N, Rutter H, Foster C (2012). Green space and physical activity: an observational study using health survey for England data. Health Place.

[55] Cockerham WC, Snead MC, DeWaal DF (2002). Health lifestyles in Russia and the socialist heritage. J Health Soc Behav.

[56] Abbott PA, Turmov S, Wallace C (2006). Health world views of post-soviet citizens. Soc Sci Med.

[57] Neufeld M, Rehm J (2013). Alcohol consumption and mortality in Russia since 2000: are there any changes following the alcohol policy changes starting in 2006?. Alcohol Alcohol.

[58] Hinote B, Cockerham WC, Abbott P (2009). Psychological distress and dietary patterns in eight post-Soviet republics. Appetite.

[59] Kiefer I, Rathmanner T, Kunze M (2005). Eating and dieting differences in men and women. J Mens Health Gend.

[60] Johnson CL, Catalano DJ (1981). Childless elderly and their family supports. Gerontologist.

[61] Fenwick R, Barresi CM (1981). Health consequences of marital-status change among the elderly: a comparison of cross-sectional and longitudinal analyses. J Health Soc Behav.

[62] Cafferata GL (1987). Marital status, living arrangements, and the use of health services by elderly persons. J Gerontol.

[63] Bonsang E (2009). Does informal care from children to their elderly parents substitute for formal care in Europe?. J Health Econ.

[64] Kay R (2013). ‘She’s like a daughter to me’: insights into care, work and kinship from rural Russia. Eur Asia Stud.

[65] Karlsen S, Nazroo JY (2002). Agency and structure: the impact of ethnic identity and racism on the health of ethnic minority people. Sociol Health Illn.

[66] Utsey SO, Chae MH, Brown CF, Kelly D (2002). Effect of ethnic group membership on ethnic identity, race-related stress and quality of life. Cultur Divers Ethnic Minor Psychol.

[67] Verkuyten M (2004). Social psychology of ethnic identity. Psychology Press.

[68] Zaridze D, Lewington S, Boroda A, Sculo G, Karpov R, Lazarev A, Konobeevskaya I, Igitov V, Terechova T, Boffetta P, Sherliker P, Kong X, Whitlock G, Boreham J, Brennan P, Peto R (2014). Alcohol and mortality in Russia: prospective observational study of 151 000 adults. Lancet.

[CR69] The pre-publication history for this paper can be accessed here: http://www.biomedcentral.com/1471-2458/14/1183/prepub

